# Printing Parameter Optimization of Additive Manufactured PLA Using Taguchi Design of Experiment

**DOI:** 10.3390/polym15224370

**Published:** 2023-11-10

**Authors:** Bilal Anjum Ahmed, Uzair Nadeem, Abbas Saeed Hakeem, Anwar Ul-Hamid, Mohd Yusuf Khan, Muhammad Younas, Hasan Aftab Saeed

**Affiliations:** 1 Core Research Facilities (CRF), Research Institute, King Fahd University of Petroleum and Minerals, Dhahran 31261, Saudi Arabia; anwar@kfupm.edu.sa (A.U.-H.); muhammad.younas@kfupm.edu.sa (M.Y.); 2 Department of Mechanical Engineering, National University of Sciences and Technology (NUST), Islamabad 44000, Pakistanhasan.saeed@ceme.nust.edu.pk (H.A.S.); 3 Interdisciplinary Center for Hydrogen Energy Storage (IRC-HES), Research Institute, King Fahd University of Petroleum and Minerals, Dhahran 31261, Saudi Arabia; ashakeem@kfupm.edu.sa (A.S.H.); mykhan@kfupm.edu.sa (M.Y.K.)

**Keywords:** fused deposition modelling, 3D printing, Taguchi optimization, tensile strength, PLA

## Abstract

Three-dimensional printing (3DP), known as additive layer manufacturing (ALM), is a manufacturing process in which a three-dimensional structure is constructed by successive addition of deposited layers. Fused Deposition Modeling (FDM) has evolved as the most frequently utilized ALM process because of its cost-effectiveness and ease of operation. Nevertheless, layer adhesion, delamination, and quality of the finished product remain issues associated with the FDM process parameters. These issues need to be addressed in order to satisfy the requirements commonly imposed by the conventional manufacturing industry. This work is focused on the optimization of the FDM process and post-process parameters for Polylactic acid (PLA) samples in an effort to maximize their tensile strength. Infill density and pattern type, layer height, and print temperature are the process parameters, while annealing temperature is the post-process parameter considered for the investigation. Analysis based on the Taguchi L18 orthogonal array shows that the gyroid infill pattern and annealing cycle at 90 °C results in a maximum ultimate tensile strength (UTM) of 37.15 MPa. Furthermore, the regression model developed for the five variables under study was able to predict the UTS with an accuracy of more than 96%.

## 1. Introduction

The future of manufacturing is being reshaped by the unprecedented growth projected for the global three-dimensional (3D) printing (3DP) industry, as indicated by Fortune’s Business Insights. With expectations of a sharp rise from USD 22.40 billion in 2021 to USD 105.99 billion by 2030 and a remarkable compound annual growth rate of 24.9%, 3D printing is poised to redefine the landscape of innovation and production [1]. The prominent feature of 3DP technology is its inherent ability to manufacture complex geometries with minimal wastage of material, along with the provision to easily adopt digital alterations in the design, which is ideal for rapid prototyping [2,3,4,5]. It has proven to be a vital component of Industry 4.0 [6,7,8,9], which continues to be an active area of research in today’s advanced manufacturing field [10,11,12,13,14,15]. Among various types of AM techniques, fused deposition modeling (FDM) is the extrusion-based type where the thermoplastic filament is extruded through a pre-heated nozzle, and the material is selectively deposited onto a build platform. The deposition of heated filament is carried out layer-by-layer, where the extruder head moves up after depositing a single layer and then deposits the subsequent layer. The process continues until the entire part is printed in this sequence. The thermoplastic filaments having a round cross-section are guided to the pre-heated extruder body employing the gear mechanism driven by a stepper motor (extruder motor). The filaments become heated up in the extruder body and pass through the brass nozzle from the lower end to be deposited on the print bed. Besides the extrusion motor, the printing path is controlled by other stepper motors that regulate the movement of the extrusion head in the three axes (*x*, *y*, *z*). The most used thermoplastic filaments in the FDM process include polylactic acid (PLA), acrylonitrile butadiene styrene (ABS), polyethylene terephthalate glycol (PETG) and polypropylene (PP) [16,17]. The ease of printing, relatively high dimensional accuracy, and environmentally friendly nature of PLA has made it the most widely employed printing material for FDM. It is important to mention that some FDM filaments require a heated print bed to achieve sufficient adhesion between the bed surface and the first printed layer. The print bed is usually kept slightly above the glass transition temperature of the filament employed to prevent the peeling effect. For instance, in the case of ABS, the bed temperature typically used is 100 °C, while for PLA, which is used in the present study, the bed temperature is set around 60 °C [17,18,19]. 

The process settings greatly influence the mechanical characteristics and geometric precision of the FDM printed object. With regard to the study on mechanical properties, frequently investigated parameters include layer height/thickness, infill density, infill percentage, build orientation, raster angle, print/extrusion temperature, and extrusion/print speed [20,21,22,23,24,25,26,27,28,29,30,31,32,33,34,35,36,37,38,39,40,41]. Infill pattern is yet another essential process parameter that has been much studied, and the diversity of the patterns reported include linear, grid, rectilinear, triangular, tri-hexagon, hexagonal, cubic, cubic sub-division, quarter cubic, cross, honeycomb, and concentric [26,32,33,35,37,39,40].

Chacón et al. studied the effect of build orientation, layer thickness, and filament feed rate on the tensile behavior and flexural strength of AM-manufactured PLA [22]. Samples developed using flat and edge build orientation (fibers aligned in the direction of applied force) resulted in better tensile strength as compared to the upright position, while the smallest layer thickness of 0.06 mm performed well in improving behavior under tensile loading. Moreover, a high feed rate of 80 mm/s resulted in better tensile strength for samples prepared using a small layer height of 0.06 mm and vice versa. Sandanamsamy et al. studied the effect of printing temperature and raster angle on the mechanical properties of FDM-printed PLA samples [23]. The highest tensile strength of 16.95 MPa was reported for the 90° raster angle and the highest nozzle temperature of 220 °C. In the study conducted by Hikmat et al. on the optimization of PLA parts using the fused deposition modeling (FDM) process, the most significant process parameters for enhancing tensile strength were identified. According to the results of variance analysis, build orientation (45%), nozzle diameter (25%), and infill percentage (19%) were found to be the key factors, highlighting their relative influence on the tensile strength of the manufactured parts. [24]. The maximum strength of 58.05 MPa was reported for the optimum parameters of (on-edge) built orientation, 30° raster orientation, 0.5 mm nozzle diameter, 220 °C extruder temperature, 100% infill density, three shell numbers, and 20 mm/s extruding speed. Zhao et al. worked on the development of tensile strength models for FDM PLA parts [25]. It was reported that the highest printing angle of 90° (fibers aligned in the direction of applied force) and lower value of layer thickness 0.1 mm results in the highest tensile strength of 49.66 MPa. Mani et al. studied the effect of various FDM process parameters on the surface and mechanical characteristics of PLA samples [29]. It was reported that a layer thickness of 0.35 mm, infill density of 65%, and nozzle temperature of 220 °C resulted in a maximum tensile strength of 44.5 MPa. Hamat et al. reported a study on the fabrication of PLA filament for the FDM process. Extrusion temperature in the range of 175–180 °C and extrusion speed in the range of 4–5 rpm resulted in smooth filament with a diameter in the range of 1.75–1.77 mm [34]. Three-dimensionally-printed parts fabricated from the extruded filament had a maximum tensile strength of 28 MPa. Öteyaka et al. investigated the effect of infill density and pattern on the flexural strength and vibrational damping of 3D printed PLA samples [37]. The tri-hexagonal infill pattern resulted in the highest flexural strength, while the infill pattern of cross-type resulted in maximum vibrational damping. Maurya et al. reported a study on the dimensional accuracy of 3D printed PLA as regards the variation in infill density and pattern [32]. A tri-hexagonal infill pattern with 20% infill resulted in maximum dimensional accuracy.

Sajjad et al. studied the combined effect of dual infill patterns in a 3D printed PLA sample on the strength-to-weight ratio and material consumption [40]. Combinations of four types of infill patterns, namely, rectangular, rectilinear, triangular, and honeycomb, were used in pairs of two. One type of pattern covered the gripping areas, while the other covered the central gauge area. The combination of rectangular and triangular infill patterns resulted in a 28% increase in the strength-to-weight ratio with 11% lesser material consumption as compared to the sample manufactured from only rectangular infill. Szust et al. reported a study on the relation between build orientation, layer height, post-printing heat treatment, and the tensile strength of FDM printed materials [27]. In the case of PLA, post-processing at 60 °C for 1 h increased the strength by 24%. However, the sample deformation during annealing was a drawback. Salt-remelting of PETG printed samples at 210 °C resulted in considerable improvement in tensile strength without having to compromise the dimensional accuracy of the samples.

Rodriguez-Reyna et al. studied the effect of infill pattern, percentage, printing direction, and layer height on the mechanical properties of 3D printed PLA, ABS, and Nylon-CF samples [33]. It was reported that the change in tensile strength due to variation in infill pattern type (linear, tridimensional, or hexagonal) and infill orientation (0°, 90°, or +45/−45°) was less significant as compared to the material selected for printing. The lower layer thickness value improved the tensile strength of the additively manufactured parts. Tandon et al. developed 3D printed tensile test samples of PLA, ABS, and PLA/CF materials having triangular infill patterns with varying degrees of infill percentages (20%, 50%, 80%) in the gauge region [41]. The build orientation varied from horizontal to inclined to vertical. Horizontal build orientation was reported to be the most desirable, having the highest strength of 24.5 MPa for PLA samples with 80% infill. FDM of PLA reinforced with wood, ceramic, copper, aluminum, and carbon fiber was studied by Liu. et al. [30]. In the upright orientation, PLA, PLA/wood, and PLA/carbon fiber had better printability as compared to ceramic and metallic reinforcements. Mechanical properties were measured to be superior for 3D printed PLA composites manufactured using metallic reinforcements. On-edge printing orientation with +45/−45° raster lay-up resulted in the highest mechanical strength. Zerankeshi et al. worked on the development of a PLA/graphene 3D printing filament by modifying the PLA surface with graphene reinforcement [36]. The compressive strength of the 3D printed PLA/1%-graphene improved from 26.71 to 38.6 MPa, while the tensile strength increased from 32.60 to 46.24 MPa.

Lokesh et al. studied the effect of layer thickness, build orientation, and raster angle on the mechanical properties of additively manufactured PLA [28]. The Taguchi L9 orthogonal array was designed to assess the effect of the process parameters. Layer thickness negatively correlating with the tensile strength was reported as the most influencing parameter. Maximum UTS of 46.65 MPa was reported for 0.1 mm layer height, 30° raster angle, and horizontal build orientation. Taguchi process parameter optimization for mechanical properties of 3D printed PLA was reported by Sahoo et al. [31]. An L9 orthogonal array of three variables, namely layer thickness, infill percentage, and print speed, each having three levels, was developed to assess the effect on tensile strength and hardness. Layer thickness and infill percentage were reported to be the significant factors. Singh et al. worked on process parameter optimization using the L27 orthogonal array for FDM of PLA samples [35]. It was reported that build orientation and infill percentage were the most significant parameters affecting tensile strength. The highest mechanical strength of 32.34 MPa was measured for samples having 80% infill, concentric infill pattern, and edge build orientation. Alafaghani et al. studied the dimensional accuracy and tensile strength of 3D printed PLA using the L9 Taguchi orthogonal array [39]. Four process parameters (infill density, infill pattern, extrusion temperature, and layer height), each having three levels, were varied. Lower values of extrusion temperature, infill density, layer height, and a hexagonal infill pattern resulted in better dimensional accuracy. In contrast, a triangular pattern with 100% infill and the highest extrusion temperature of 210 °C with 0.3 mm layer thickness resulted in maximum tensile strength. Vidakis et al. studied the quality of 3D printed ABS samples (surface roughness, porosity, and geometric accuracy) using an L25 Taguchi orthogonal array with six parameters, each having five levels [38]. All six control parameters (raster angle, infill density, nozzle temperature, bed temperature, print speed, and layer thickness) were reported to affect the final print quality significantly. Furthermore, predictive mathematical models for surface roughness, porosity, and dimensional accuracy were developed and validated. Durga et al. studied the effect of layer thickness, extrusion temperature, and infill pattern type on the tensile strength of PLA/carbon fiber composite by an L27 Taguchi orthogonal array for the design of the experiment (DOE) [26]. The lowest layer thickness of 0.1 mm, highest extrusion temperature of 225 °C, and cubic infill pattern resulted in the optimal tensile strength of 26.59 MPa.

Optimization of mechanical properties and geometric accuracy of FDM PLA parts by varying the process parameters have been extensively reported in the literature. However, it was identified that the Gyroid and Octet 3D infill patterns have not been studied much. Furthermore, little attention has been paid to the post-printing heat treatment of 3D printed parts. To the best of our knowledge, no work has been reported on the optimization of printing parameters coupled with post-printing heat treatment for FDM PLA samples. Hence, this study aims to optimize the tensile properties of 3D printed PLA samples by identifying the most favorable combination of the four most crucial printing parameters (layer height, infill pattern, infill percentage, printing temperature) along with the post-printing heat treatment temperature.

## 2. Experimental Procedure

In the present study, CREALITY Ender 3 v2, having a print build size of 220 × 220 × 250 mm, was used to manufacture the PLA tensile samples additively. The material used for this study is commercial green color PLA, 1.75 mm in diameter, acquired from SUNLU. PLA is a thermoplastic polymer created from renewable resources, such as corn starch, sugarcane, or cassava.

The variable process parameters were the infill density, layer thickness, infill pattern type, nozzle temperature, and annealing temperature. Different levels of these process parameters are tabulated in Table 1. In Table 1, the infill pattern types are denoted as follows: level 1 for the Octet infill pattern, level 2 for the Tri-hexagonal infill pattern, and level 3 for the Gyroid infill pattern. Moreover, printing speed, print bed temperature, build orientation, and raster angle were the fixed printing parameters for all the samples. Default values, as defined by Ultimaker Cura 4.13.1 (slicing software), are employed for the number of inner and outer layers and shell thickness. The values of the fixed printing parameters are listed in Table 2.

The complete factorial design for four variables having three levels and one variable having two levels would mean running a total of 162 experiments (3^4^ × 2^1^), which is not a very feasible option. Therefore, in order to reduce the number of experiments, a Taguchi L18 orthogonal array was created using MINITAB 19 software. The designed L18 orthogonal array presented in Table 3 is then used to additively manufacture the samples.

**Figure 1:**
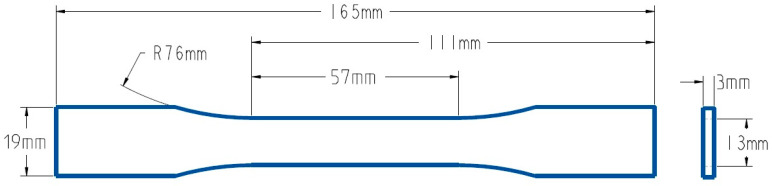
ASTM 638D Type I standard specimen drawing for tensile testing.

**Figure 2:**
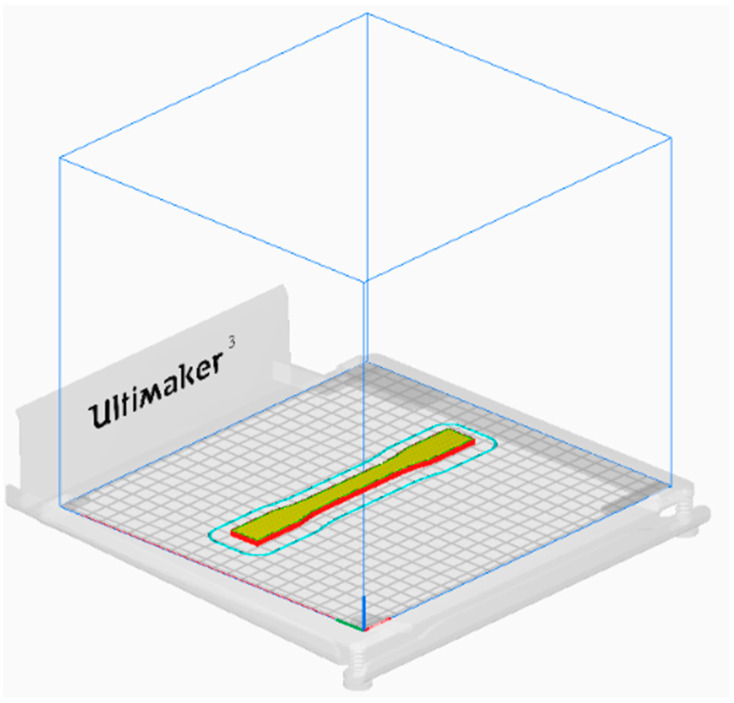
Flat orientation of the tensile sample imported and placed in Ultimaker Cura.

**Figure 3:**
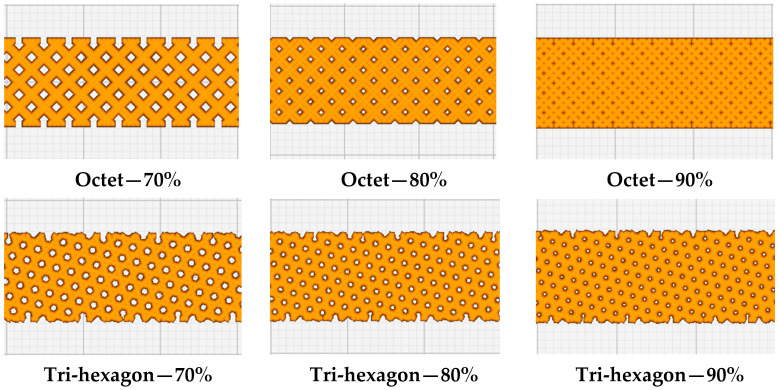

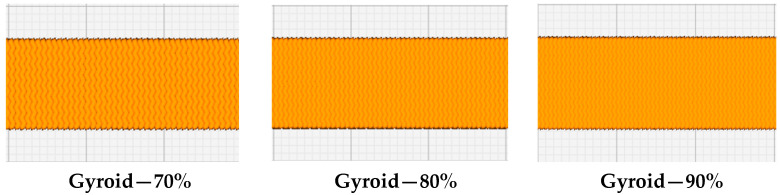
The three infill patterns employed with infill percentages varying from 70% to 90%.

In order to assess the tensile characteristics, all the samples were prepared according to the ASTM 638D [42] Type I standard for tensile testing (Figure 1). The 3D geometry of the tensile specimen generated using SolidWorks 2021 modeling software in line with the ASTM standard is then converted into STL format and imported to the slicing software Ultimaker Cura. Figure 2 shows the orientation (flat) of the sample after importing it to Ultimaker Cura. Three-dimensional printable g-code files were then developed in the Ultimaker Cura software according to the printing parameters of the individual samples (as defined in Table 3). The infill patterns employed in the fabrication of tensile samples, along with the infill percentages, are depicted in Figure 3. 

**Figure 4:**
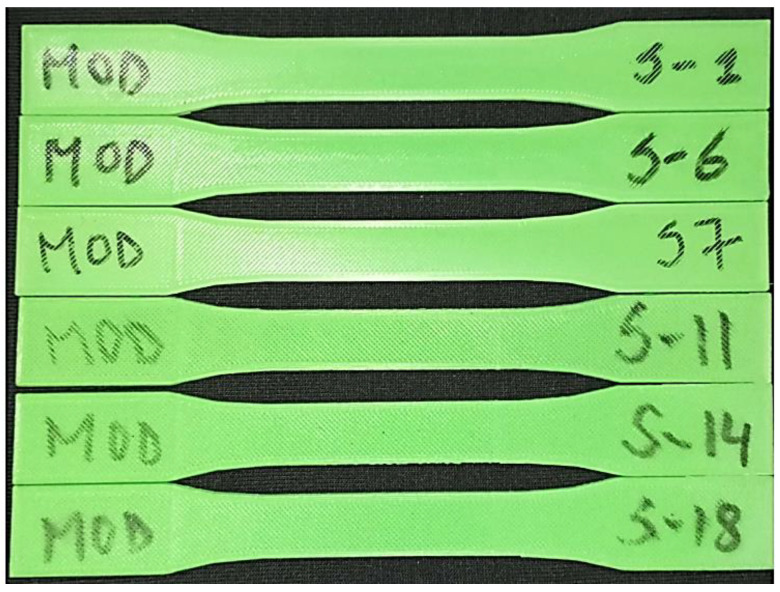
As-printed FDM PLA samples are not subject to annealing heat treatment.

**Figure 5:**
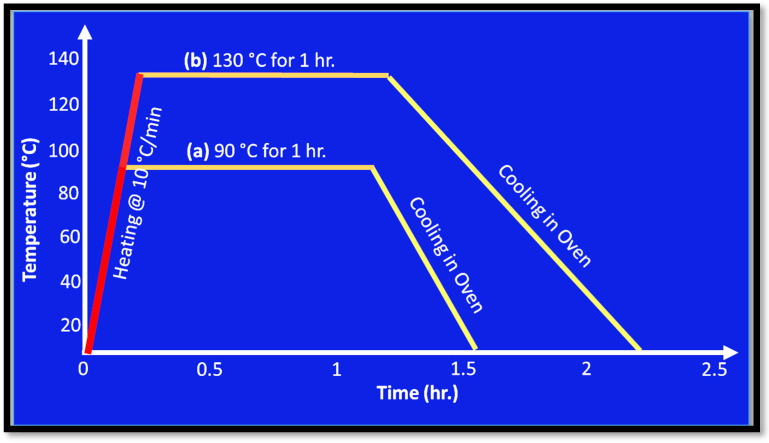
(a) Annealing cycle for samples no. 2, 4, 8, 12, 15, 16 and (b) annealing cycle for samples no. 3, 5, 9, 10, 13, 17.

Some of the 3D printed samples used are shown in Figure 4. Post-printing annealing of the selected samples was carried out at 90 and 130 °C. Annealing PLA involves heating the PLA specimen above the glass transition temperature (around 60 °C for PLA), holding the specimens isothermally at the designed temperature for a predetermined time, and cooling the samples within the oven/furnace. Annealing can be performed in many ways, primarily using a heat gun, oven, or furnace. In this study, annealing is conducted using a drying oven. As specified in Table 3, samples No. 2, 4, 8, 12, 15, and 16 were annealed at 90 °C for one hour, whereas samples No. 3, 5, 9, 10, 13, and 17 were annealed at 130 °C for the same duration. The annealing cycle for the aforementioned samples is represented in Figure 5. 

Tensile testing of samples was performed using a Shimadzu universal testing machine (UTM) with a 1 KN load cell and a crosshead speed of 1 mm/min. All tests were performed at room temperature condition. 

The tensile testing data were further processed using Minitab statistical software to identify the optimum processing parameters, validate the maximum tensile strength attained in light of the optimized process parameters, and develop a regression equation for UTS based on the process parameters. In order to better understand the mechanical behavior of the tested samples, the fracture surface of selected samples was observed under the JEOL JSM 6610LV scanning electron microscope (SEM). The samples were gold coated using the Cressington 108 Auto Sputter Coater for 20 s prior to the SEM examination. Moreover, selected samples were characterized using a Rigaku Ultima IV X-ray diffractometer to analyze the effect of heat treatment on the crystallinity of the samples and to correlate it with the mechanical behavior.

## 3. Results and Discussion

### 3.1. Tensile Test Results of Taguchi Matrix

**Figure 6:**
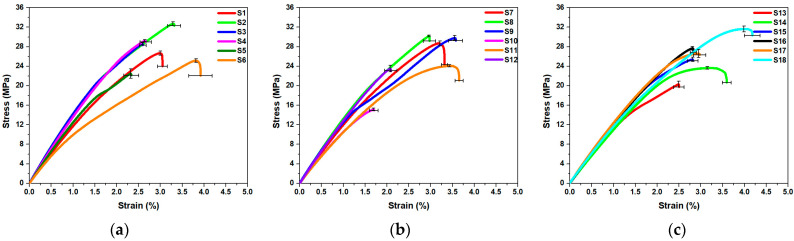
Stress–strain graphs for the samples (**a**) S1–S6, (**b**) S7–S12, and (**c**) S13–S18.

The tensile stress–strain plots for each sample in the Taguchi L18 orthogonal array are presented in Figure 6a–c. Ultimate tensile strength and percentage elongation for all the samples are tabulated in Table 4. It is observed that sample number 18 has the highest ultimate tensile strength, while sample number 10 has the lowest UTS. Sample 18 was observed to have the maximum ductility, while sample 10 was measured to have the lowest. 

### 3.2. Signal/Noise Ratio Analysis

The effect of FDM process parameters and the annealing treatment on the mechanical properties was assessed by computing the signal-to-noise (S/N) ratio for every sample in the orthogonal L18 Taguchi array (as shown in Table 5). The S/N ratio calculated using Minitab software facilitates data analysis and identifies the optimum values of process parameters for the desired output. In the present work, the larger-the-better criterion was selected to maximize the UTS and percentage elongation. 

**Figure 7:**
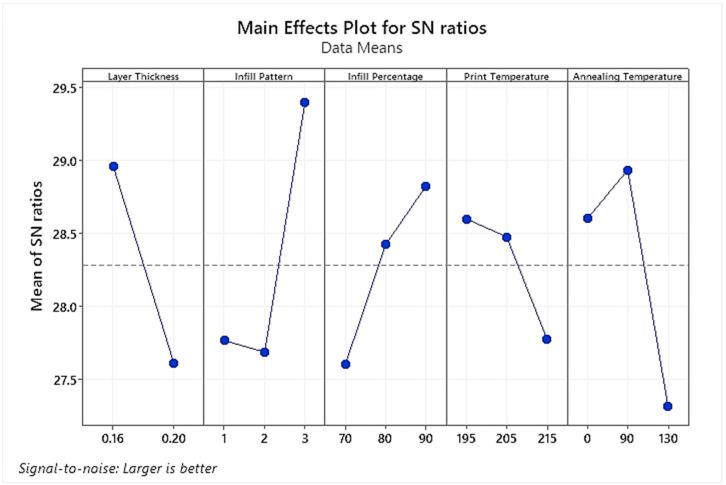
Plot of S/N ratios for UTS mean responses.

The main effect plot for the UTS means S/N ratio values as generated by the statistical software is represented in Figure 7. Table 6 shows the corresponding values for mean S/N ratios and the maximum effectiveness rank for each processing factor. These ranks are calculated based on delta values for each factor, where the delta represents the variance/scatter between the highest and lowest average response for the respective processing factor.

It is evident from Figure 7 that printing temperature and layer thickness have a negative correlation with the UTS. Therefore, it is clearly inferred that a lower value of layer thickness (0.16 mm) and minimum printing temperature of 195 °C would optimize the UTS. Contrary to this, the infill percentage was seen to have a direct relation with the increase in UTS. Moreover, a Gyroid-type infill pattern (3) and annealing at a moderate temperature of 90 °C were observed to be the most suitable levels for the two processing factors. Furthermore, it is evident from Table 6 that the infill pattern having the highest delta value is the most significant processing parameter, followed by the annealing temperature, while the printing temperature was measured to be the least influential of the five processing parameters.

**Figure 8:**
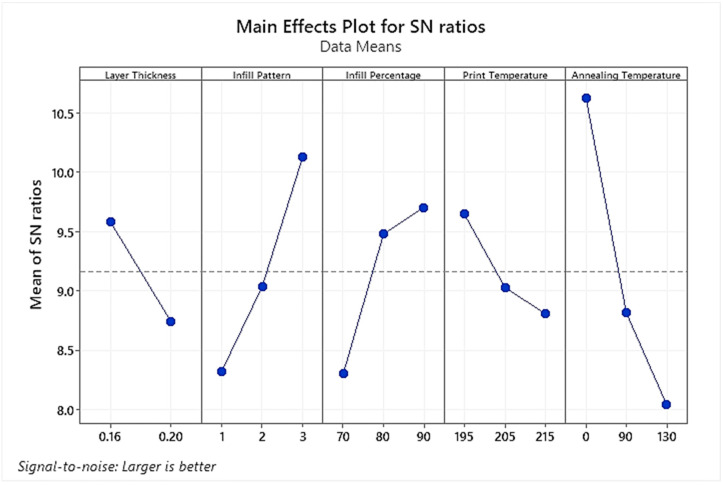
Plot of S/N ratios for % Elongation mean responses.

The main effect plot for the % Elongation mean S/N ratio values as generated by the statistical software is represented in Figure 8. Table 7 shows the corresponding values for mean S/N ratios and the maximum effectiveness ranks for each processing factor. In light of the S/N ratio plot, it is evident that lower layer thickness of 0.16 mm, Gyroid-type infill pattern, highest infill density of 90%, lowest printing temperature of 195 °C and as printed sample (without annealing) are the optimized processing parameters required to achieve maximum ductility. Barkhad et al., in their research on the annealing treatment of melt-extruded PLA, have also revealed that it significantly increased the PLA’s stiffness and compressive strength while reducing its ductility [43]. Consequently, annealing was the most influential process parameter affecting the ductility, followed by the infill pattern. Similar to the case of UTS, printing temperature was observed to be the least significant process parameter affecting the sample’s ductility.

**Figure 9:**
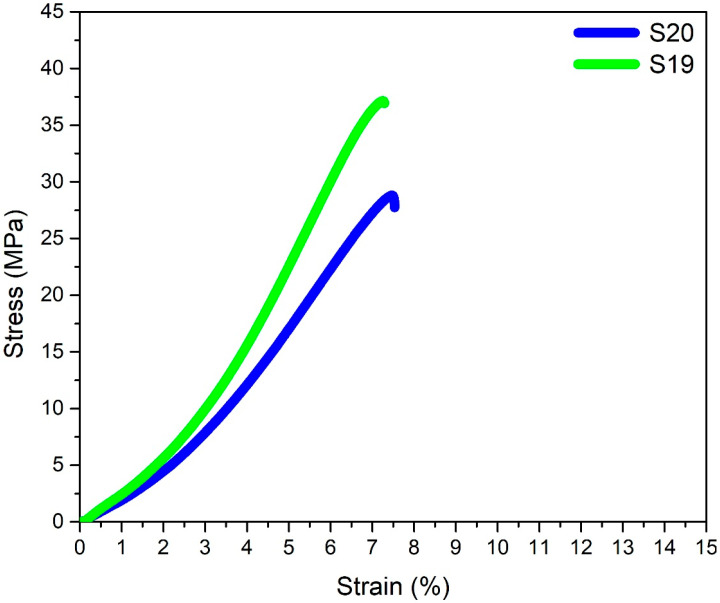
Stress–strain graphs for the samples S19 and S20.

In light of the tensile test results of the L18 orthogonal array, the maximum tensile strength was measured to be 34.6 MPa, and the highest % Elongation was 4.0% for the sample S18. With reference to S/N ratio plots, the optimized process parameters for UTS and % Elongation are tabulated in Table 8. However, samples using these conditions were not part of the original L18 orthogonal design array. Therefore, the next step was to conduct confirmatory tests using the best parameters, i.e., S19 and S20. The tensile stress/strain plots of the two samples are presented in Figure 9. Tensile tests on sample S19 resulted in a maximum UTS value of 37.15 MPa. Similarly, sample S20 was the most ductile sample, with a strain percentage value of 7.53%. These samples were later used to verify the accuracy of regression models developed (in the next section) for forecasting UTS and % Elongation based on the process parameters. 

### 3.3. Regression Models

The regression equation aims to understand the response behavior by changing the process parameters. The added advantage of the regression equation is to make predictions based on the initial process parameters. The regression fit equations for UTS were developed using the three types of infill patterns as the categorical variables. In contrast, layer thickness, infill percentage, print temperature, and annealing temperatures were chosen as the continuous variables. The experimental values of UTS for the samples in L18 orthogonal were used to develop the three models. Validation of the model, represented by Equation (3), was performed using the process parameters of the sample S19. The predicted value of 35.79 MPa against the experimental value of 37.15 MPa validated the suitability and effectiveness of the regression model. The regression models represented below, having an R^2^ value of 72.46%, provide a sound base for further experimentation. It could be further improved by increasing the sample size for each type of infill pattern.

Infill Pattern: Octet

(1) UTSMPa=49.1−91.1LayerThickness+0.1608InfillPercentage−0.0958PrintingTemperature+0.0932Annealing Temperature−0.000914 Annealing Temperature2

Infill Pattern: Tri-hexagonal

(2) UTSMPa=48.3−91.1LayerThickness+0.1608InfillPercentage−0.0958PrintingTemperature+0.0932Annealing Temperature−0.000914 Annealing Temperature2

Infill Pattern: Gyroid

(3) UTSMPa=53.6−91.1LayerThickness+0.1608InfillPercentage−0.0958PrintingTemperature+0.0932Annealing Temperature−0.000914 Annealing Temperature2

### 3.4. Structural Characterization 

In light of the Taguchi analysis, infill pattern type and annealing temperature were seen to be the most significant parameters affecting the tensile strength of FDM printed samples. In order to better understand the effect of the aforementioned parameters, selected samples were analyzed using scanning electron microscopy and X-ray diffraction. The fracture surface of sample S10, having the lowest tensile strength of 15.1 MPa, and sample S19, having the highest tensile strength of 37.15 MPa, were selected for observation under SEM. In comparison, the samples S19 and S20 were selected for the XRD to assess the effect of annealing temperature on the crystallinity of the samples and, therefore, on the tensile behavior. 

**Figure 10:**
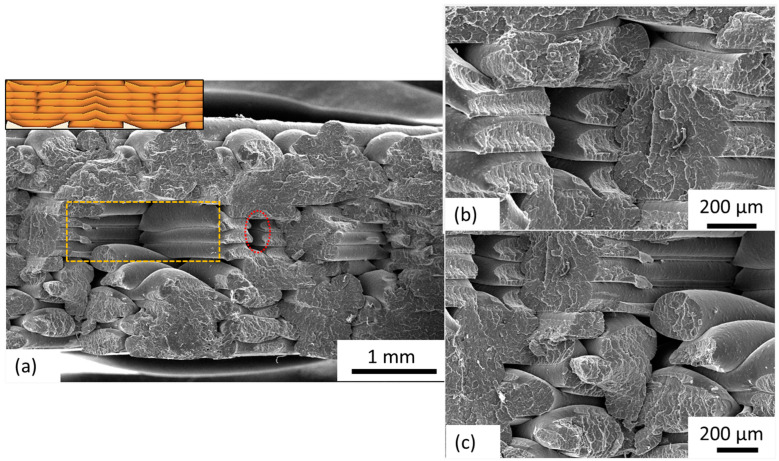
SEM images acquired from the fractured surface of sample S10 where (**a**) represents the surface at low magnification; inset depicts the cross-sectional view of the deposited layers; (**b**,**c**) represent selective regions at high magnification; widening of the gap between layers and individual fiber displacement and fracture is evident.

Figure 10a shows the fracture surface SEM image acquired from sample S10. The inset represents the cross-sectional view of the layers for sample S10. The triangular porous pockets (black region) are the inherent limitation of the geometry. The region highlighted in the red ellipse and the corresponding high magnification SEM image (Figure 10b) represents a clear widening of the triangular porous pockets upon application of tensile loading, indicating poor intra-layer bonding. The region highlighted using a yellow rectangle corresponds to the V-shaped wedge visible in the inset. The absence of material from this region (due to the Octet infill design) results in failure/fracture of individual fibers under overload. Furthermore, the displacement of individual fibers and their fracture, as shown in Figure 10b,c, indicates poor fiber adhesion within and between the layers.

**Figure 11:**
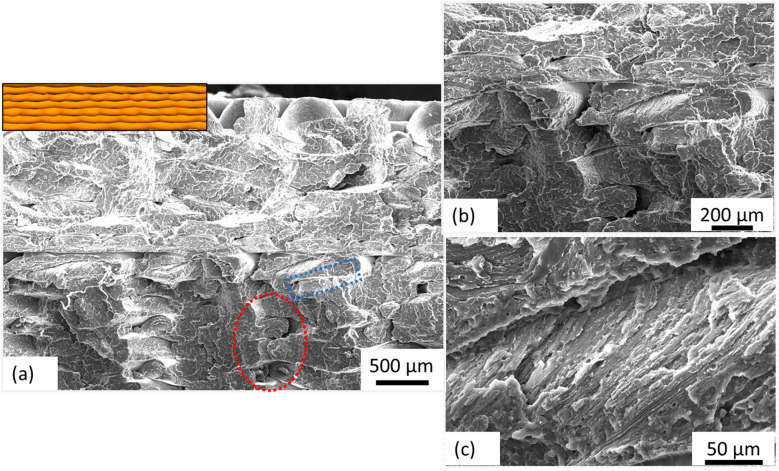
SEM images acquired from the fractured surface of sample S19 where (**a**) represents the surface at low magnification; inset depicts the cross-sectional view of the deposited layers; (**b**,**c**) represents selective regions at high magnification; better interlocking between and within layers is evident.

Figure 11a represents the fracture surface SEM image of sample S19, and the inset represents the cross-sectional view of the layer-by-layer deposition for the same sample (Gyroid infill pattern). The inherent signs of porosity and resultant separation of fibers are not observed. The in-plane and out-of-plane propagation of the crack (the encircled region in red color), as represented in a high magnification image in Figure 11b, explains better adhesion between the intra-layer fibers. Figure 11c represents the high magnification SEM image of the inter-layer region highlighted blue in Figure 11a. It is observed that fibers within consecutive layers are better adhered as compared to inter and intra-layer separation observed in sample S10.

**Figure 12:**
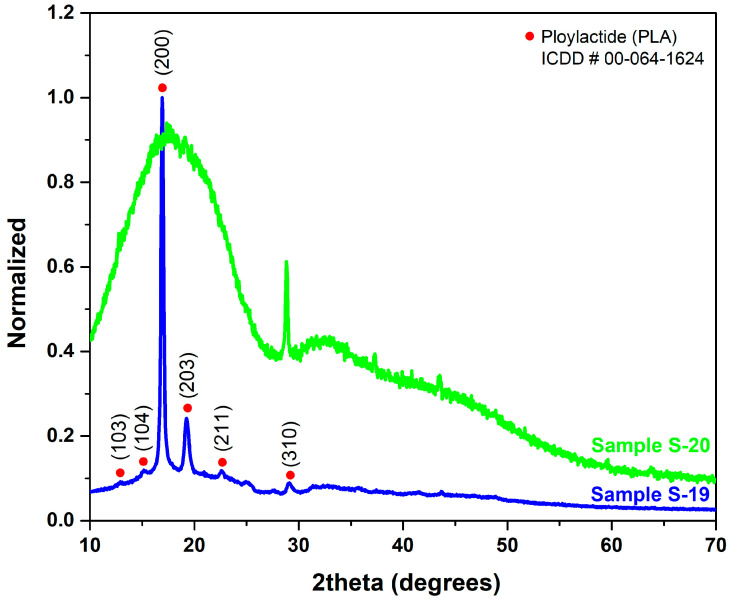
XRD spectrums were acquired from samples subjected to the same printing parameters with (S19) and without (S20) heat-treatment.

By employing wide-angle X-ray diffraction in the 2-theta range 10° to 70°, the effect of annealing temperature on the crystallinity of 3D printed samples was identified. Figure 12 represents the XRD spectra of samples S19 and S20 as it is seen that the as-printed sample (S20) contains an amorphous ‘halo’ with only one crystalline peak from the (310) plane. In contrast, sample S19 represents a semi-crystalline sample with PLA chains crystallized in the α-form orthorhombic crystals. The diffraction spectrum shows multiple crystalline peaks from (103), (104), (200), (203), (211), and (310) planes. The X-ray diffraction analysis reveals a degree of crystallinity (Xc) of 40.46% for sample S19 and 6.63% for sample S20, as calculated from the WAXD patterns. Annealing of the printed sample (S19) at 90 °C for 1 h of isothermal treatment significantly enhances the crystallinity of the sample, which positively enhances the tensile strength of the sample. Brian et al. made a similar observation, reporting high-intensity XRD reflections at (200) and (203) during the annealing of PLA at 100 °C due to the transition from the less stable α phase to the more stable α phase within the temperature range of 90–120 °C [44]. This enhancement was linked to the increase in percentage crystallinity at higher annealing temperatures.

## 4. Conclusions

This study highlights the novel approach of employing Taguchi statistical analysis to optimize both process and post-process parameters for maximizing the tensile strength of 3D printed PLA samples. Among various FDM printing process parameters, the research focused on analyzing the critical variables, namely ‘layer height’, ‘infill pattern’, ‘infill density’, and ‘print temperature’. Additionally, the study incorporated ‘annealing temperature’ as a crucial post-processing parameter. Through the identification of these key parameters, development of regression models, and correlation analyses, the following conclusions are made:

- Signal-to-noise ratio analysis showed that maximum tensile strength could be achieved with 0.16 mm layer height, 90% infill density, Gyroid-type infill pattern, 195 °C print temperature, and 90 °C annealing temperature. The maximum UTS value, as predicted by the regression model, was 35.79 MPa;
- Maximum tensile strength of 37.15 MPa was measured for the FDM sample developed using the optimized parameters. The developed regression model under-predicted the maximum UTS with a reasonably acceptable deviation of 3.6%;
- Infill pattern and annealing temperature were identified as the most significant processing parameters. Octet infill pattern resulting in porous V-shaped wedges proved to be detrimental against tensile loading, whereas the Gyroid type having a wavy infill pattern resulted in much better inter and intra-layer adhesion, thereby imparting superior mechanical properties;
- Annealing at 90 °C for 1 h resulted in improved UTS compared to the as-printed (non-heat-treated) sample. Crystallographic analysis revealed the transformation of the amorphous structure of as-printed PLA samples to semi-crystalline orthorhombic α-form when subjected to annealing at 90 °C. The enhanced crystallinity is believed to have significantly contributed towards a higher UTS.

## Figures and Tables

**Table 1:** Process parameters and their corresponding level values.

Process Parameters	Level I	Level II	Level III
**Layer thickness (mm)**	0.16	0.20	-
Infill Pattern *	1	2	3
Infill Percentage (%)	70	80	90
Printing Temperature (°C)	195	205	215
Annealing Temperature (°C)	0	90	130

* 1 = Octet, 2 = Tri-hexagonal, 3 = Gyroid.

**Table 2:** Fixed process parameters employed during 3D printing.

Process Parameter	Parameter Value Set
Print Speed	50 mm/s
Bed Temperature	60 °C
Build Orientation	Flat/Horizontal
Raster Angle	90°
Top and Bottom Layers thickness	0.8 mm
Shell thickness	0.8 mm
Line width	0.4 mm

**Table 3:** L18 orthogonal array for the design of experiments.

Sample No.	Layer Thickness (mm)	Infill Pattern 1 = Octet, 2 = Tri-hexagonal, 3 = Gyroid	Infill Percentage (%)	Print Temperature (°C)	Annealing Temperature (°C)
S1	0.16	1	70	195	0
S2	0.16	1	80	205	90
S3	0.16	1	90	215	130
S4	0.16	2	70	195	90
S5	0.16	2	80	205	130
S6	0.16	2	90	215	0
S7	0.16	3	70	205	0
S8	0.16	3	80	215	90
S9	0.16	3	90	195	130
S10	0.20	1	70	215	130
S11	0.20	1	80	195	0
S12	0.20	1	90	205	90
S13	0.20	2	70	205	130
S14	0.20	2	80	215	0
S15	0.20	2	90	195	90
S16	0.20	3	70	215	90
S17	0.20	3	80	195	130
S18	0.20	3	90	205	0

**Table 4:** Ultimate tensile strength and percentage elongation of the samples.

Sample No.	Ultimate Tensile Strength MPa					Percentage Elongation%				
	1	2	3	AV	SD	1	2	3	AV	SD
S1	26.7	26.2	27.1	26.66	(0.45)	3.0	3.1	2.9	3.00	(0.11)
S2	32.7	33.1	32.3	32.68	(0.40)	3.3	3.1	3.4	3.27	(0.15)
S3	28.6	28.4	28.8	28.58	(0.23)	2.6	2.5	2.7	2.58	(0.08)
S4	29.2	29.2	29.6	29.32	(0.20)	2.7	2.6	2.8	2.69	(0.13)
S5	22.5	21.5	23.4	22.47	(0.95)	2.3	2.2	2.5	2.32	(0.17)
S6	25.1	24.9	25.6	25.22	(0.36)	3.8	3.6	4.1	3.85	(0.27)
S7	28.7	28.2	29.2	28.68	(0.53)	3.2	3.2	3.3	3.23	(0.07)
S8	30.2	30.0	30.3	30.19	(0.17)	3.0	3.1	2.9	2.98	(0.14)
S9	29.8	29.3	30.2	29.78	(0.47)	3.5	3.3	3.6	3.48	(0.16)
S10	15.1	14.8	15.3	15.08	(0.25)	1.7	1.6	1.8	1.68	(0.10)
S11	24.1	24.3	23.9	24.08	(0.23)	3.4	3.3	3.5	3.38	(0.09)
S12	23.5	22.9	24.2	23.54	(0.65)	2.1	2.2	1.9	2.07	(0.14)
S13	20.3	19.7	20.9	20.31	(0.60)	2.5	2.4	2.6	2.50	(0.12)
S14	23.6	23.3	23.8	23.56	(0.28)	3.6	3.5	3.7	3.60	(0.10)
S15	25.4	25.0	25.8	25.38	(0.43)	2.8	2.7	3.0	2.83	(0.12)
S16	27.7	27.3	28.0	27.66	(0.35)	2.8	2.7	2.9	2.82	(0.07)
S17	26.6	25.8	27.4	26.60	(0.80)	3.0	2.7	3.0	2.90	(0.14)
S18	31.6	30.9	32.1	31.56	(0.61)	4.2	3.9	4.1	4.05	(0.18)

**Table 5:** Signal/noise ratios for UTS and % Elongation calculated using Minitab software.

Sample No.	UTS (MPa)	S/N Ratio	% Elongation	S/N Ratio
S1	26.7	28.53	3.0	9.54
S2	32.7	30.29	3.3	10.37
S3	28.6	29.13	2.6	8.30
S4	29.2	29.34	2.7	8.63
S5	22.5	27.04	2.3	7.23
S6	25.1	28.03	3.8	11.60
S7	28.7	29.16	3.2	10.10
S8	30.2	29.60	3.0	9.54
S9	29.8	29.48	3.5	10.88
S10	15.1	23.58	1.7	4.61
S11	24.1	27.64	3.4	10.63
S12	23.5	27.42	2.1	6.44
S13	20.3	26.15	2.5	7.96
S14	23.6	27.46	3.6	9.83
S15	25.4	28.10	2.8	8.94
S16	27.7	28.85	2.8	8.94
S17	26.6	28.50	3.0	9.25
S18	31.6	30.78	4.2	12.04

**Table 6:** S/N ratio analysis for UTS and effectiveness rank of process parameters.

Process Parameters	S/N Ratio			Delta	Effectiveness Rank
	Level 1	Level 2	Level 3		
Layer Thickness	28.19	27.61	-	1.35	3
Infill Pattern	27.76	27.76	27.76	27.76	1
Infill Percentage	27.6	27.6	27.6	27.6	4
Print Temperature	28.6	28.6	28.6	28.6	5
Annealing Temperature	28.6	28.6	28.6	28.6	2
Criterion: Larger is Better					

**Table 7:** S/N ratio analysis for %-elongation and effectiveness rank of process parameters.

Process Parameters	S/N Ratio			Delta	Effectiveness Rank
	Level 1	Level 2	Level 3		
Layer Thickness	9.577	8.738	-	0.839	5
Infill Pattern	8.316	9.031	10.127	1.811	2
Infill Percentage	8.297	9.475	9.701	1.404	3
Print Temperature	9.645	9.025	8.803	0.842	4
Annealing Temperature	10.623	8.812	8.039	2.585	1
Criterion: Larger is Better					

**Table 8:** Optimized Processing Parameters for maximum UTS and % Elongation.

**UTS Optimized Parameters**					
**Sample No.**	**Layer Thickness (mm)**	**Infill Pattern** **3 = Gyroid**	**Infill Percentage (%)**	**Print Temperature (°C)**	**Annealing Temperature (°C)**
S19	0.16	3	90	195	90
**% Elongation Optimized Parameters**					
**Sample No.**	**Layer Thickness (mm)**	**Infill Pattern** **3 = Gyroid**	**Infill Percentage (%)**	**Print Temperature (°C)**	**Annealing Temperature (°C)**
S20	0.16	3	90	195	0

## Data Availability

Data is contained within the article.
